# ELEMENTS OF DIGNITY FROM THE VIEWPOINT OF STAFF IN LONG TERM CARE FACILITIES AND THE INTERACTION DIGNITY MODEL

**DOI:** 10.1093/geroni/igae098.3964

**Published:** 2024-12-31

**Authors:** Nanako Hasegawa, Yukari Niimi, Chikako Sone, Eriko Otake, Cynthia Jacelon

**Affiliations:** University of Massachusetts Amherst, Amherst, Massachusetts, United States; Nagoya Women’s University, Nagoya, Aichi, Japan; Nagano College of Nursing, Komagane, Nagano, Japan; National College of Nursing, Japan, Kiyose, Tokyo, Japan; University of Massachusetts Amherst, Amherst, Massachusetts, United States

## Abstract

Japan has the highest proportion of older people of any country in the world. Culturally, it is important that older adults who enter long term care (LTC) facilities maintain their dignity as they did in their previous lives. However, providing dignified is challenging. Lack of dignity in care threatens not only the dignity and health of residents, but also that of staff. To contextualize and visualize elements of dignity from the perspective of staff, and using a hermeneutic phenomenological prospective approach, we conducted individual and follow-up interviews (18 interviews total) with 9 staff members (4 nurses and 5 certified care workers; age 25–43) working in LTC. Interviews were recorded and transcribed verbatim. Codes were extracted and organized into subthemes and themes. Member checking, blind checking, and a panel of seven judges were used to reduce bias and improve the validity of the findings. Two themes emerged as the core of individual dignity: “Individual dignity not affected by others” and “The individual dignity of the staff.” Three themes were identified as representing the interactions between staff and society: “Dignified care in the narrow sense,” “Dignified care by LTC facilities and the LTC system,” and “Dignity in relation to family members, friends, society, and other residents”. Identifying the “individual dignity of the staff” was identified as a significant finding. Dignity in LTC facilities includes not only dignity of residents but also of the staff, and “Dignified care in the narrow sense” was identified in the interaction between these two.

